# Crystal structure of ({4-[(4-bromo­phen­yl)ethyn­yl]-3,5-di­ethyl­phen­yl}ethyn­yl)triiso­propyl­silane

**DOI:** 10.1107/S2056989015007252

**Published:** 2015-04-18

**Authors:** Caiyun Shu, Graeme J. Moxey, Adam Barlow, Mahbod Morshedi

**Affiliations:** a School of Chemical and Material Engineering, Jiangnan University, Wuxi 214122, People’s Republic of China; b Research School of Chemistry, Australian National University, Canberra, ACT 2601, Australia

**Keywords:** crystal structure, tri­alkyl­silyl­acetyl­ene, bromo­arene, oligo(phenyl­eneethynylene)

## Abstract

The title compound, C_29_H_37_BrSi, was synthesized by the Sonogashira coupling of [(3,5-diethyl-4-ethynylphen­yl)ethyn­yl]triiso­propyl­silane with 4-bromo-1-iodo­benzene. In the structure, the two phenyl rings are nearly parallel to each other with a dihedral angle of 4.27 (4)°. In the crystal, π–π inter­actions between the terminal and central phenyl rings of adjacent mol­ecules link them in the *a*-axis direction [perpendicular distance = 3.5135 (14); centroid–centroid distance = 3.7393 (11) Å]. In addition, there are weak C—H⋯π inter­actions between the isopropyl H atoms and the phenyl rings of adjacent mol­ecules.

## Related literature   

For the syntheses of aryl­alkynes by Sonogashira coupling, see: Takahashi *et al.* (1980 ▸). For the use of related oligo(phenyl­eneethynylene)s in the construction of metal alkynyl complexes exhibiting non-linear optical properties, see: Garcia *et al.* (2002 ▸); Hurst *et al.* (2002 ▸; 2003 ▸); McDonagh *et al.* (2003 ▸). For the synthesis of [(3,5-diethyl-4-iodo­phen­yl)ethyn­yl]triiso­propyl­silane, see: Ehlers *et al.* (2011 ▸). For related structures, see: Lehnherr *et al.* (2008 ▸, 2009 ▸); Błaszczyk *et al.* (2007 ▸).
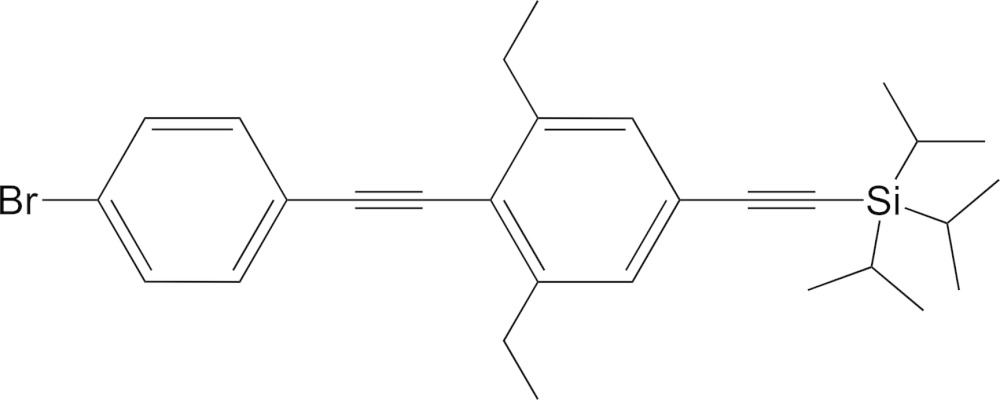


## Experimental   

### Crystal data  

C_29_H_37_BrSi
*M* *_r_* = 493.58
Monoclinic, 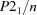

*a* = 14.9043 (2) Å
*b* = 8.50185 (11) Å
*c* = 22.6111 (3) Å
β = 108.2791 (16)°
*V* = 2720.56 (7) Å^3^
*Z* = 4
Cu *K*α radiation
μ = 2.56 mm^−1^
*T* = 150 K
0.19 × 0.06 × 0.05 mm

### Data collection  

Agilent SuperNova (Dual, Cu at zero, EosS2) diffractometer
Absorption correction: analytical [*CrysAlis PRO* (Agilent, 2014 ▸), based on expressions derived by Clark & Reid (1995 ▸)] *T*_min_ = 0.910, *T*_max_ = 0.973
17549 measured reflections
5355 independent reflections
4677 reflections with *I* > 2σ(*I*)
*R*_int_ = 0.030

### Refinement  

*R*[*F* ^2^ > 2σ(*F* ^2^)] = 0.036
*wR*(*F* ^2^) = 0.093
*S* = 1.03
5355 reflections
288 parameters
H-atom parameters constrained
Δρ_max_ = 0.44 e Å^−3^
Δρ_min_ = −0.64 e Å^−3^

### 

Data collection: *CrysAlis PRO* (Agilent, 2014 ▸); cell refinement: *CrysAlis PRO*; data reduction: *CrysAlis PRO*; program(s) used to solve structure: *SHELXS97* (Sheldrick, 2008 ▸); program(s) used to refine structure: *SHELXL2013* (Sheldrick, 2015 ▸); molecular graphics: *OLEX2* (Dolomanov *et al.*, 2009 ▸); software used to prepare material for publication: *OLEX2*.

## Supplementary Material

Crystal structure: contains datablock(s) I. DOI: 10.1107/S2056989015007252/hg5438sup1.cif

Structure factors: contains datablock(s) I. DOI: 10.1107/S2056989015007252/hg5438Isup2.hkl

Supporting information file. DOI: 10.1107/S2056989015007252/hg5438Isup3.cml

**Fig. 1.**
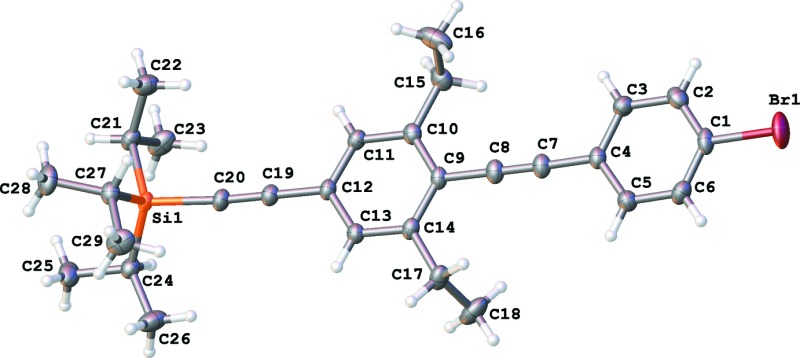
Molecular structure of ((4-((4-bromophenyl)ethynyl)-3,5-diethylphenyl)ethynyl)triisopropylsilane, with thermal ellipsoids set at the 40% probability level.

. DOI: 10.1107/S2056989015007252/hg5438fig1.tif
Mol­ecular structure of ((4-((4-bromo­phen­yl)ethyn­yl)-3,5-di­ethyl­phen­yl)ethyn­yl)triiso­propyl­silane, with thermal ellipsoids set at the 40% probability level.

**Fig. 2.**
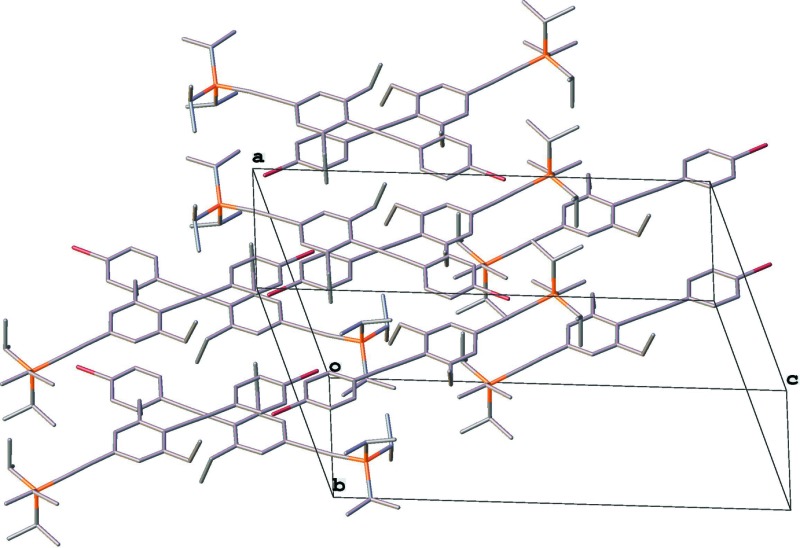
Packing diagram of ((4-((4-bromophenyl)ethynyl)-3,5-diethylphenyl)ethynyl)triisopropylsilane.

. DOI: 10.1107/S2056989015007252/hg5438fig2.tif
Packing diagram of ((4-((4-bromo­phen­yl)ethyn­yl)-3,5-di­ethyl­phen­yl)ethyn­yl)triiso­propyl­silane.

**Fig. 3.**
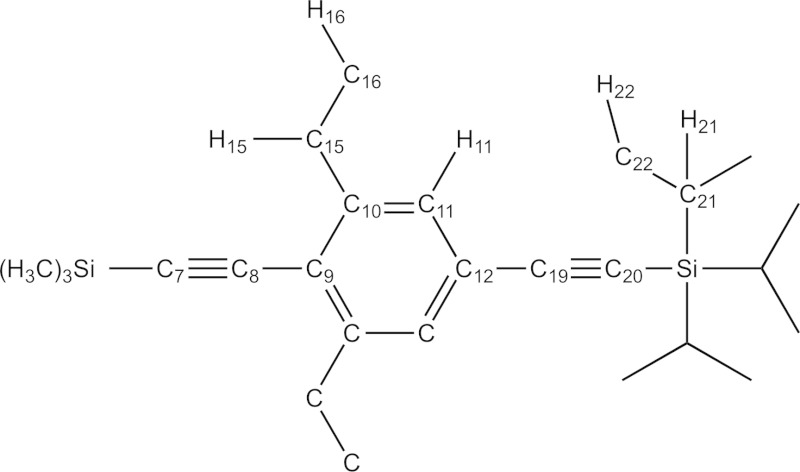
Atom numbering scheme of ((2,6-diethyl-4-((triisopropylsilyl)ethynyl)phenyl)ethynyl)trimethylsilane for 1H and 13C NMR assignments.

1 13 . DOI: 10.1107/S2056989015007252/hg5438fig3.tif
Atom numbering scheme of ((2,6-diethyl-4-((triiso­propyl­sil­yl)ethyn­yl)phen­yl)ethyn­yl)tri­methyl­silane for ^1^H and ^13^C NMR assignments.

**Fig. 4.**
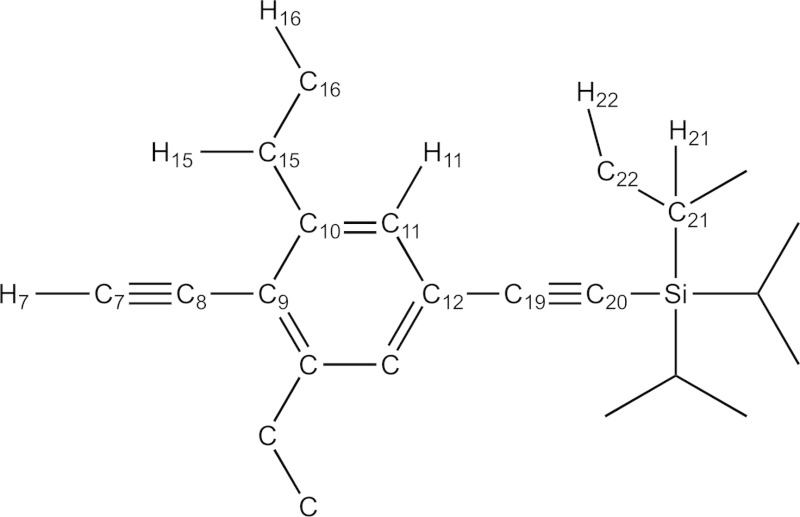
Atom numbering scheme of ((3,5-diethyl-4-ethynylphenyl)ethynyl)triisopropylsilane for 1H and 13C NMR assignments.

1 13 . DOI: 10.1107/S2056989015007252/hg5438fig4.tif
Atom numbering scheme of ((3,5-diethyl-4-ethynylphen­yl)ethyn­yl)triiso­propyl­silane for ^1^H and ^13^C NMR assignments.

**Fig. 5.**
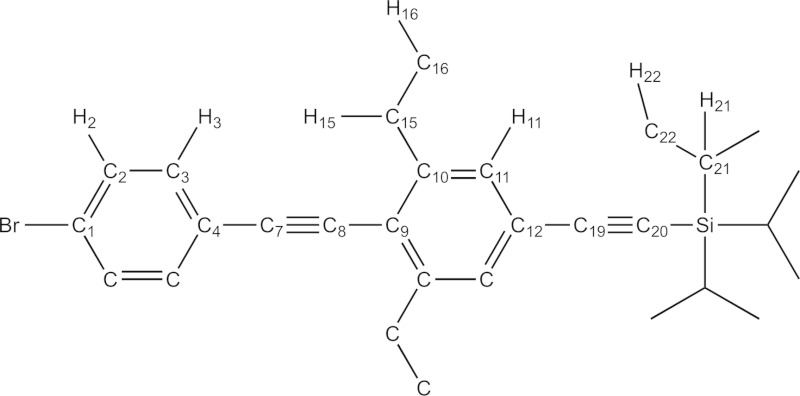
Atom numbering scheme of ((4-((4-bromophenyl)ethynyl)-3,5-diethylphenyl)ethynyl)triisopropylsilane for 1H and 13C NMR assignments.

1 13 . DOI: 10.1107/S2056989015007252/hg5438fig5.tif
Atom numbering scheme of ((4-((4-bromo­phen­yl)ethyn­yl)-3,5-di­ethyl­phen­yl)ethyn­yl)triiso­propyl­silane for ^1^H and ^13^C NMR assignments.

CCDC reference: 1059001

Additional supporting information:  crystallographic information; 3D view; checkCIF report

## Figures and Tables

**Table 1:** Hydrogen-bond geometry (, ) *Cg* is the centroid of the C9C14 ring.

*D*H*A*	*D*H	H*A*	*D* *A*	*D*H*A*
C25H25*B* *Cg* ^i^	0.96	2.98	3.699(3)	132

Symmetry code: (i) 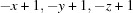.

## supplementary crystallographic information

### S1. Synthesis and crystallization

As described herein, the title compound was prepared in three steps from ((3,5-di­ethyl-4-iodo­phenyl)­ethynyl)triiso­propyl­silane; the synthesis of ((3,5-di­ethyl-4-iodo­phenyl)­ethynyl)triiso­propyl­silane is described in: Ehlers *et al.* (2011).

*1. Synthesis of ((2,6-di­ethyl-4-((triiso­propyl­silyl)ethynyl)phenyl)­ethynyl)tri­methyl­silane*

((3,5-Di­ethyl-4-iodo­phenyl)­ethynyl)triiso­propyl­silane (325 mg, 0.740 mmol) was added to tri­ethyl­amine (15 mL) and the solvent was de­oxy­genated. Triiso­propyl­silyl­acetyl­ene (0.2 mL, 1.45 mmol) was then added, followed by Pd(PPh_3_)_4_ (30 mg, 0.025 mmol) and CuI (5.0 mg, 0.025 mmol) and the mixture was stirred at room temperature for 24 h. The solvent was removed under reduced pressure and the residue was purified using column chromatography on silica, eluting with petrol. The solvent was removed from the eluate to give

((2,6-di­ethyl-4-((triiso­propyl­silyl)ethynyl)phenyl)­ethynyl)tri­methyl­silane as a pale yellow liquid (0.194 g, 64%). ^1^H NMR (δ, 400MHz, CDCl_3_): 0.26 (s, 9H, H_(Si(CH3)3_), 1.13 (s, 21H, H_21_, H_22_), 1.23 (t, J_HH_ = 7.5Hz, 6H, H_16_), 2.77 (q, J_HH_ = 7.5Hz, 4H, H_15_), 7.14 (s, 2H, H_11_). ^13^C NMR (δ, 101MHz, CDCl_3_): 146.9 (C_10_), 128.9 (C_11_), 123.1 (C_9_), 121.9 (C_12_), 107.4 (C_19_), 103.6 (C_8_), 102.0 (C_7_), 91.4 (C_20_), 28.0 (C_15_), 18.8 (C_22_), 14.5 (C_16_), 11.4 (C_21_), 0.12 (C_(SiCH3)3_). MS—EI: *m/z* (fragment, relative intensity): 410.2 ([M]^+^, 8).

*2. Synthesis of ((3,5-di­ethyl-4-ethynyl­phenyl)­ethynyl)triiso­propyl­silane*

((2,6-Di­ethyl-4-((triiso­propyl­silyl)ethynyl)phenyl)­ethynyl)tri­methyl­silane (0.947 g, 2.31 mmol) was added to a mixture of THF and ethanol (1:1, 50 mL). An aqueous solution of NaOH (2.5 mL, 0.1 M) was then added, and the mixture was stirred for 30 min. The solvent was removed under reduced pressure and the residue was purified using column chromatography on silica, eluting with petrol. The solvent was removed to give ((3,5-di­ethyl-4-ethynyl­phenyl)­ethynyl)triiso­propyl­silane as a pale yellow liquid (0.706 g, 90%). ^1^H NMR (δ, 400MHz, CDCl_3_): 1.13 (s, 21H, H_21_, H_22_), 1.24 (t, J_HH_ = 7.5Hz, 6H, H_16_), 2.80 (q, J_HH_ = 7.5Hz, 4H, H_15_), 3.50 (s, 1H, H_7_), 7.17 (s, 2H, H_11_). ^13^C NMR (δ, 101MHz, CDCl_3_): 147.2 (C_10_), 128.9 (C_11_), 123.5 (C_9_), 120.7 (C_12_), 107.2 (C_19_), 91.6 (C_20_), 85.8 (C_7_), 80.3 (C_8_), 27.8 (C_15_), 18.7 (C_22_), 14.7 (C_16_), 11.4 (C_21_). MS—EI: *m/z* (fragment, relative intensity): 338.3 ([M]^+^, 26).

*3. Synthesis of ((4-((4-bromo­phenyl)­ethynyl)-3,5-di­ethyl­phenyl)­ethynyl)triiso­propyl­silane*

((3,5-Di­ethyl-4-ethynyl­phenyl)­ethynyl)triiso­propyl­silane (0.140 g, 0.415 mmol) and 4-bromo-1-iodo­benzene (0.139 g, 0.491 mmol) was added to de­oxy­genated tri­ethyl­amine (40 mL). PdCl_2_(PPh_3_)_2_ (9.0 mg, 0.12 mmol) and CuI (4 mg, 0.02 mmol) were then added, and the resultant solution was stirred at room temperature for 16 h. The solvent was then removed under vacuum and the residue was passed through a silica column, eluting with petrol. The solvent was reduced in volume to give ((4-((4-bromo­phenyl)­ethynyl)-3,5-di­ethyl­phenyl)­ethynyl)triiso­propyl­silane as a white solid (0.192 g, 96%). Anal. Calc. for C_29_H_37_BrSi: C, 70.57; H, 7.56. Found: C, 70.53; H, 7.61%. ^1^H NMR (δ, 400MHz, CDCl_3_): 1.14 (s, 21H, H_21_, H_22_), 1.28 (t, J_HH_ = 7.5Hz, 6H, H_16_), 2.84 (q, J_HH_ = 7.5Hz, 4H, H_15_), 7.20 (s, 2H, H_11_), 7.37 (d, J_HH_ = 7.5Hz, 2H, H_3_), 7.49 (d, J_HH_ = 7.5Hz, 2H, H_2_). ^13^C NMR (δ, 101MHz, CDCl_3_): 146.9 (C_10_), 132.8 (C_3_), 131.8 (C_2_), 128.9 (C_11_), 123.1 (C_9_), 122.8 (C_1 or 4_), 122.6 (C_1 or 4_), 121.9 (C_12_), 107.4 (C_19_), 97.2 (C_7_), 91.9 (C_20_), 87.8 (C_8_), 28.1 (C_15_), 18.8 (C_22_), 14.8 (C_16_), 11.5 (C_21_). MS—EI: *m/z* (fragment, relative intensity): 494.2 ([M]^+^, 10). Colorless crystals of the title compound were obtained by slow evaporation of a hexane solution at room temperature.

### S2. Refinement

Crystal data, data collection and structure refinement details are summarized below.

### Crystal data

**Table:**

C_29_H_37_BrSi	*F*(000) = 1040
*M**_r_* = 493.58	*D*_x_ = 1.205 Mg m^−^^3^
Monoclinic, *P*2_1_/*n*	Cu *K*α radiation, λ = 1.54184 Å
*a* = 14.9043 (2) Å	Cell parameters from 7858 reflections
*b* = 8.50185 (11) Å	θ = 3.1–72.1°
*c* = 22.6111 (3) Å	µ = 2.56 mm^−^^1^
β = 108.2791 (16)°	*T* = 150 K
*V* = 2720.56 (7) Å^3^	Needle, colorless
*Z* = 4	0.19 × 0.06 × 0.05 mm

### Data collection

**Table:**

Agilent SuperNova (Dual, Cu at zero, EosS2) diffractometer	5355 independent reflections
Radiation source: sealed X-ray tube, SuperNova (Cu) X-ray Source	4677 reflections with *I* > 2σ(*I*)
Mirror monochromator	*R*_int_ = 0.030
Detector resolution: 8.1297 pixels mm^-1^	θ_max_ = 72.3°, θ_min_ = 4.1°
ω scans	*h* = −17→18
Absorption correction: analytical [*CrysAlis PRO* (Agilent, 2014), based on expressions derived by Clark & Reid (1995)]	*k* = −10→8
*T*_min_ = 0.910, *T*_max_ = 0.973	*l* = −26→27
17549 measured reflections	

### Refinement

**Table:**

Refinement on *F*^2^	Primary atom site location: structure-invariant direct methods
Least-squares matrix: full	Hydrogen site location: inferred from neighbouring sites
*R*[*F*^2^ > 2σ(*F*^2^)] = 0.036	H-atom parameters constrained
*wR*(*F*^2^) = 0.093	*w* = 1/[σ^2^(*F*_o_^2^) + (0.0487*P*)^2^ + 0.7988*P*] where *P* = (*F*_o_^2^ + 2*F*_c_^2^)/3
*S* = 1.03	(Δ/σ)_max_ = 0.001
5355 reflections	Δρ_max_ = 0.44 e Å^−^^3^
288 parameters	Δρ_min_ = −0.64 e Å^−^^3^
0 restraints	

### Special details

**Table:**

Experimental. Absorption correction: CrysAlisPro (Agilent Technologies, 2014) Analytical numeric absorption correction using a multifaceted crystal model based on expressions derived by R.C. Clark & J.S. Reid. (Clark & Reid, 1995). Empirical absorption correction using spherical harmonics, implemented in SCALE3 ABSPACK scaling algorithm.
Geometry. All e.s.d.'s (except the e.s.d. in the dihedral angle between two l.s. planes) are estimated using the full covariance matrix. The cell e.s.d.'s are taken into account individually in the estimation of e.s.d.'s in distances, angles and torsion angles; correlations between e.s.d.'s in cell parameters are only used when they are defined by crystal symmetry. An approximate (isotropic) treatment of cell e.s.d.'s is used for estimating e.s.d.'s involving l.s. planes.

### Fractional atomic coordinates and isotropic or equivalent isotropic displacement parameters (Å^2^)

**Table:**

	*x*	*y*	*z*	*U*_iso_*/*U*_eq_	
Br1	0.59936 (2)	1.35829 (4)	−0.03406 (2)	0.06342 (11)	
C1	0.60740 (12)	1.2473 (2)	0.04038 (8)	0.0367 (4)	
C2	0.61900 (13)	1.3304 (2)	0.09424 (10)	0.0391 (4)	
H2	0.6229	1.4396	0.0944	0.047*	
C3	0.62480 (13)	1.2492 (2)	0.14846 (8)	0.0353 (4)	
H3	0.6346	1.3043	0.1855	0.042*	
C4	0.61611 (11)	1.0861 (2)	0.14807 (8)	0.0297 (3)	
C5	0.60401 (13)	1.0054 (2)	0.09239 (8)	0.0351 (4)	
H5	0.5979	0.8965	0.0914	0.042*	
C6	0.60100 (13)	1.0858 (3)	0.03850 (8)	0.0386 (4)	
H6	0.5948	1.0316	0.0017	0.046*	
C7	0.61969 (12)	1.0040 (2)	0.20410 (8)	0.0346 (4)	
C8	0.62174 (12)	0.9394 (2)	0.25178 (8)	0.0340 (3)	
C9	0.62603 (11)	0.8617 (2)	0.30887 (7)	0.0285 (3)	
C10	0.63335 (11)	0.9506 (2)	0.36276 (8)	0.0303 (3)	
C11	0.64252 (11)	0.8724 (2)	0.41843 (7)	0.0296 (3)	
H11	0.6479	0.9302	0.4543	0.036*	
C12	0.64376 (11)	0.7080 (2)	0.42108 (7)	0.0277 (3)	
C13	0.63343 (11)	0.62185 (19)	0.36677 (7)	0.0284 (3)	
H13	0.6324	0.5126	0.3683	0.034*	
C14	0.62465 (11)	0.6958 (2)	0.31058 (7)	0.0287 (3)	
C15	0.63495 (14)	1.1285 (2)	0.36204 (10)	0.0396 (4)	
H15A	0.6087	1.1683	0.3933	0.048*	
H15B	0.5955	1.1657	0.3217	0.048*	
C16	0.73415 (18)	1.1921 (3)	0.37476 (16)	0.0639 (7)	
H16A	0.7619	1.1475	0.3456	0.096*	
H16B	0.7717	1.1647	0.4164	0.096*	
H16C	0.7318	1.3045	0.3704	0.096*	
C17	0.61268 (14)	0.5983 (2)	0.25298 (8)	0.0384 (4)	
H17A	0.6384	0.4941	0.2652	0.046*	
H17B	0.6483	0.6460	0.2284	0.046*	
C18	0.51011 (17)	0.5835 (3)	0.21337 (10)	0.0575 (6)	
H18A	0.5062	0.5266	0.1760	0.086*	
H18B	0.4835	0.6865	0.2028	0.086*	
H18C	0.4756	0.5281	0.2363	0.086*	
C19	0.65751 (12)	0.6270 (2)	0.47885 (8)	0.0306 (3)	
C20	0.67199 (12)	0.5571 (2)	0.52729 (7)	0.0318 (3)	
C21	0.68643 (12)	0.6214 (2)	0.65936 (8)	0.0330 (3)	
H21	0.7019	0.5752	0.7011	0.040*	
C22	0.75465 (17)	0.7576 (3)	0.66248 (10)	0.0482 (5)	
H22A	0.8184	0.7191	0.6762	0.072*	
H22B	0.7469	0.8353	0.6913	0.072*	
H22C	0.7415	0.8040	0.6219	0.072*	
C23	0.58501 (15)	0.6822 (3)	0.64139 (11)	0.0497 (5)	
H23A	0.5668	0.7226	0.5996	0.075*	
H23B	0.5810	0.7645	0.6695	0.075*	
H23C	0.5435	0.5978	0.6437	0.075*	
C24	0.61059 (12)	0.3018 (2)	0.59826 (8)	0.0328 (3)	
H24	0.5494	0.3552	0.5885	0.039*	
C25	0.62337 (16)	0.2156 (3)	0.65974 (10)	0.0493 (5)	
H25A	0.6800	0.1535	0.6700	0.074*	
H25B	0.5700	0.1482	0.6556	0.074*	
H25C	0.6281	0.2910	0.6922	0.074*	
C26	0.60039 (18)	0.1852 (3)	0.54504 (11)	0.0554 (5)	
H26A	0.5933	0.2418	0.5071	0.083*	
H26B	0.5458	0.1204	0.5402	0.083*	
H26C	0.6557	0.1201	0.5544	0.083*	
C27	0.82830 (12)	0.3998 (2)	0.62548 (8)	0.0337 (3)	
H27	0.8635	0.4924	0.6193	0.040*	
C28	0.87126 (14)	0.3534 (3)	0.69440 (10)	0.0492 (5)	
H28A	0.8649	0.4395	0.7203	0.074*	
H28B	0.9370	0.3288	0.7029	0.074*	
H28C	0.8387	0.2631	0.7030	0.074*	
C29	0.84528 (15)	0.2720 (3)	0.58276 (11)	0.0511 (5)	
H29A	0.8215	0.1734	0.5923	0.077*	
H29B	0.9118	0.2629	0.5890	0.077*	
H29C	0.8132	0.2993	0.5402	0.077*	
Si1	0.70023 (3)	0.46269 (5)	0.60435 (2)	0.02571 (10)	

### Atomic displacement parameters (Å^2^)

**Table:**

	*U*^11^	*U*^22^	*U*^33^	*U*^12^	*U*^13^	*U*^23^
Br1	0.05680 (15)	0.0898 (2)	0.04728 (14)	0.00897 (12)	0.02157 (11)	0.04038 (12)
C1	0.0294 (8)	0.0511 (10)	0.0307 (8)	0.0024 (7)	0.0110 (6)	0.0173 (7)
C2	0.0355 (9)	0.0348 (8)	0.0469 (10)	−0.0030 (7)	0.0126 (8)	0.0087 (7)
C3	0.0375 (9)	0.0374 (8)	0.0305 (8)	−0.0025 (7)	0.0101 (7)	−0.0016 (7)
C4	0.0252 (7)	0.0369 (8)	0.0270 (7)	0.0022 (6)	0.0081 (6)	0.0071 (6)
C5	0.0374 (9)	0.0331 (8)	0.0341 (8)	0.0014 (7)	0.0103 (7)	0.0034 (7)
C6	0.0385 (9)	0.0505 (10)	0.0259 (8)	0.0016 (8)	0.0089 (7)	−0.0008 (7)
C7	0.0304 (8)	0.0430 (9)	0.0311 (8)	0.0053 (7)	0.0108 (6)	0.0084 (7)
C8	0.0294 (8)	0.0423 (9)	0.0311 (8)	0.0058 (6)	0.0106 (6)	0.0082 (7)
C9	0.0236 (7)	0.0379 (8)	0.0251 (7)	0.0052 (6)	0.0091 (6)	0.0076 (6)
C10	0.0253 (7)	0.0344 (8)	0.0310 (8)	0.0034 (6)	0.0084 (6)	0.0041 (6)
C11	0.0274 (7)	0.0367 (8)	0.0241 (7)	0.0018 (6)	0.0071 (6)	−0.0010 (6)
C12	0.0238 (7)	0.0367 (8)	0.0223 (7)	0.0014 (6)	0.0068 (5)	0.0042 (6)
C13	0.0266 (7)	0.0317 (7)	0.0265 (7)	0.0019 (6)	0.0078 (6)	0.0033 (6)
C14	0.0257 (7)	0.0376 (8)	0.0232 (7)	0.0056 (6)	0.0080 (6)	0.0021 (6)
C15	0.0400 (9)	0.0349 (9)	0.0448 (10)	0.0074 (7)	0.0147 (8)	0.0049 (7)
C16	0.0482 (12)	0.0308 (9)	0.113 (2)	0.0018 (8)	0.0260 (13)	0.0018 (11)
C17	0.0439 (10)	0.0457 (9)	0.0265 (8)	0.0110 (8)	0.0124 (7)	−0.0001 (7)
C18	0.0504 (12)	0.0779 (16)	0.0364 (10)	0.0063 (11)	0.0022 (9)	−0.0193 (10)
C19	0.0286 (7)	0.0383 (8)	0.0250 (8)	0.0004 (6)	0.0084 (6)	0.0027 (6)
C20	0.0317 (8)	0.0408 (9)	0.0226 (7)	0.0018 (6)	0.0080 (6)	0.0030 (6)
C21	0.0332 (8)	0.0415 (9)	0.0244 (7)	0.0021 (7)	0.0091 (6)	−0.0030 (6)
C22	0.0523 (11)	0.0461 (10)	0.0464 (11)	−0.0083 (9)	0.0159 (9)	−0.0115 (8)
C23	0.0391 (10)	0.0623 (12)	0.0471 (11)	0.0094 (9)	0.0127 (8)	−0.0152 (9)
C24	0.0288 (8)	0.0401 (8)	0.0284 (8)	−0.0024 (6)	0.0073 (6)	0.0009 (7)
C25	0.0481 (11)	0.0572 (12)	0.0403 (10)	−0.0159 (9)	0.0107 (8)	0.0119 (9)
C26	0.0571 (13)	0.0613 (13)	0.0480 (11)	−0.0208 (11)	0.0169 (10)	−0.0201 (10)
C27	0.0272 (7)	0.0417 (9)	0.0322 (8)	0.0025 (6)	0.0096 (6)	0.0055 (7)
C28	0.0305 (9)	0.0717 (14)	0.0406 (10)	0.0066 (9)	0.0043 (8)	0.0166 (9)
C29	0.0380 (10)	0.0573 (12)	0.0595 (13)	0.0094 (9)	0.0172 (9)	−0.0093 (10)
Si1	0.0248 (2)	0.0339 (2)	0.01806 (18)	0.00090 (15)	0.00617 (14)	0.00271 (15)

### Geometric parameters (Å, º)

**Table:**

Br1—C1	1.9003 (16)	C18—H18B	0.9600
C1—C2	1.371 (3)	C18—H18C	0.9600
C1—C6	1.376 (3)	C19—C20	1.204 (3)
C2—H2	0.9300	C20—Si1	1.8431 (17)
C2—C3	1.386 (3)	C21—H21	0.9800
C3—H3	0.9300	C21—C22	1.528 (3)
C3—C4	1.393 (3)	C21—C23	1.527 (3)
C4—C5	1.395 (3)	C21—Si1	1.8894 (17)
C4—C7	1.433 (2)	C22—H22A	0.9600
C5—H5	0.9300	C22—H22B	0.9600
C5—C6	1.386 (3)	C22—H22C	0.9600
C6—H6	0.9300	C23—H23A	0.9600
C7—C8	1.202 (3)	C23—H23B	0.9600
C8—C9	1.434 (2)	C23—H23C	0.9600
C9—C10	1.409 (2)	C24—H24	0.9800
C9—C14	1.411 (2)	C24—C25	1.530 (2)
C10—C11	1.392 (2)	C24—C26	1.530 (3)
C10—C15	1.513 (2)	C24—Si1	1.8866 (17)
C11—H11	0.9300	C25—H25A	0.9600
C11—C12	1.399 (2)	C25—H25B	0.9600
C12—C13	1.396 (2)	C25—H25C	0.9600
C12—C19	1.434 (2)	C26—H26A	0.9600
C13—H13	0.9300	C26—H26B	0.9600
C13—C14	1.387 (2)	C26—H26C	0.9600
C14—C17	1.507 (2)	C27—H27	0.9800
C15—H15A	0.9700	C27—C28	1.539 (2)
C15—H15B	0.9700	C27—C29	1.527 (3)
C15—C16	1.515 (3)	C27—Si1	1.8936 (17)
C16—H16A	0.9600	C28—H28A	0.9600
C16—H16B	0.9600	C28—H28B	0.9600
C16—H16C	0.9600	C28—H28C	0.9600
C17—H17A	0.9700	C29—H29A	0.9600
C17—H17B	0.9700	C29—H29B	0.9600
C17—C18	1.515 (3)	C29—H29C	0.9600
C18—H18A	0.9600		
			
C2—C1—Br1	119.11 (15)	C20—C19—C12	177.76 (18)
C2—C1—C6	121.98 (16)	C19—C20—Si1	175.58 (16)
C6—C1—Br1	118.90 (15)	C22—C21—H21	107.8
C1—C2—H2	120.5	C22—C21—Si1	111.30 (13)
C1—C2—C3	119.02 (17)	C23—C21—H21	107.8
C3—C2—H2	120.5	C23—C21—C22	110.13 (18)
C2—C3—H3	119.7	C23—C21—Si1	111.70 (13)
C2—C3—C4	120.68 (17)	Si1—C21—H21	107.8
C4—C3—H3	119.7	C21—C22—H22A	109.5
C3—C4—C5	118.74 (16)	C21—C22—H22B	109.5
C3—C4—C7	120.14 (17)	C21—C22—H22C	109.5
C5—C4—C7	121.12 (17)	H22A—C22—H22B	109.5
C4—C5—H5	119.7	H22A—C22—H22C	109.5
C6—C5—C4	120.69 (17)	H22B—C22—H22C	109.5
C6—C5—H5	119.7	C21—C23—H23A	109.5
C1—C6—C5	118.83 (17)	C21—C23—H23B	109.5
C1—C6—H6	120.6	C21—C23—H23C	109.5
C5—C6—H6	120.6	H23A—C23—H23B	109.5
C8—C7—C4	177.9 (2)	H23A—C23—H23C	109.5
C7—C8—C9	178.92 (18)	H23B—C23—H23C	109.5
C10—C9—C8	120.05 (16)	C25—C24—H24	105.5
C10—C9—C14	120.69 (15)	C25—C24—Si1	113.43 (12)
C14—C9—C8	119.24 (16)	C26—C24—H24	105.5
C9—C10—C15	121.63 (16)	C26—C24—C25	110.96 (18)
C11—C10—C9	119.01 (15)	C26—C24—Si1	114.88 (14)
C11—C10—C15	119.33 (16)	Si1—C24—H24	105.5
C10—C11—H11	119.6	C24—C25—H25A	109.5
C10—C11—C12	120.81 (15)	C24—C25—H25B	109.5
C12—C11—H11	119.6	C24—C25—H25C	109.5
C11—C12—C19	121.02 (15)	H25A—C25—H25B	109.5
C13—C12—C11	119.35 (15)	H25A—C25—H25C	109.5
C13—C12—C19	119.61 (15)	H25B—C25—H25C	109.5
C12—C13—H13	119.3	C24—C26—H26A	109.5
C14—C13—C12	121.37 (15)	C24—C26—H26B	109.5
C14—C13—H13	119.3	C24—C26—H26C	109.5
C9—C14—C17	121.68 (15)	H26A—C26—H26B	109.5
C13—C14—C9	118.71 (15)	H26A—C26—H26C	109.5
C13—C14—C17	119.60 (16)	H26B—C26—H26C	109.5
C10—C15—H15A	109.2	C28—C27—H27	106.2
C10—C15—H15B	109.2	C28—C27—Si1	113.21 (13)
C10—C15—C16	111.88 (16)	C29—C27—H27	106.2
H15A—C15—H15B	107.9	C29—C27—C28	111.08 (18)
C16—C15—H15A	109.2	C29—C27—Si1	113.30 (13)
C16—C15—H15B	109.2	Si1—C27—H27	106.2
C15—C16—H16A	109.5	C27—C28—H28A	109.5
C15—C16—H16B	109.5	C27—C28—H28B	109.5
C15—C16—H16C	109.5	C27—C28—H28C	109.5
H16A—C16—H16B	109.5	H28A—C28—H28B	109.5
H16A—C16—H16C	109.5	H28A—C28—H28C	109.5
H16B—C16—H16C	109.5	H28B—C28—H28C	109.5
C14—C17—H17A	109.1	C27—C29—H29A	109.5
C14—C17—H17B	109.1	C27—C29—H29B	109.5
C14—C17—C18	112.37 (16)	C27—C29—H29C	109.5
H17A—C17—H17B	107.9	H29A—C29—H29B	109.5
C18—C17—H17A	109.1	H29A—C29—H29C	109.5
C18—C17—H17B	109.1	H29B—C29—H29C	109.5
C17—C18—H18A	109.5	C20—Si1—C21	105.76 (8)
C17—C18—H18B	109.5	C20—Si1—C24	107.47 (8)
C17—C18—H18C	109.5	C20—Si1—C27	105.95 (8)
H18A—C18—H18B	109.5	C21—Si1—C27	110.17 (8)
H18A—C18—H18C	109.5	C24—Si1—C21	110.17 (8)
H18B—C18—H18C	109.5	C24—Si1—C27	116.63 (8)
			
Br1—C1—C2—C3	179.97 (14)	C12—C13—C14—C17	−179.40 (15)
Br1—C1—C6—C5	−178.08 (14)	C13—C14—C17—C18	97.4 (2)
C1—C2—C3—C4	−2.0 (3)	C14—C9—C10—C11	2.2 (2)
C2—C1—C6—C5	1.7 (3)	C14—C9—C10—C15	180.00 (15)
C2—C3—C4—C5	1.7 (3)	C15—C10—C11—C12	−178.35 (15)
C2—C3—C4—C7	−178.31 (17)	C19—C12—C13—C14	−176.79 (15)
C3—C4—C5—C6	0.2 (3)	C22—C21—Si1—C20	−61.07 (15)
C4—C5—C6—C1	−1.9 (3)	C22—C21—Si1—C24	−176.92 (13)
C6—C1—C2—C3	0.2 (3)	C22—C21—Si1—C27	53.01 (15)
C7—C4—C5—C6	−179.76 (16)	C23—C21—Si1—C20	62.49 (16)
C8—C9—C10—C11	−176.57 (15)	C23—C21—Si1—C24	−53.36 (17)
C8—C9—C10—C15	1.2 (2)	C23—C21—Si1—C27	176.56 (15)
C8—C9—C14—C13	176.92 (15)	C25—C24—Si1—C20	−178.21 (15)
C8—C9—C14—C17	−3.9 (2)	C25—C24—Si1—C21	−63.44 (17)
C9—C10—C11—C12	−0.5 (2)	C25—C24—Si1—C27	63.09 (17)
C9—C10—C15—C16	−87.6 (2)	C26—C24—Si1—C20	52.68 (17)
C9—C14—C17—C18	−81.8 (2)	C26—C24—Si1—C21	167.46 (15)
C10—C9—C14—C13	−1.9 (2)	C26—C24—Si1—C27	−66.01 (17)
C10—C9—C14—C17	177.33 (15)	C28—C27—Si1—C20	166.62 (15)
C10—C11—C12—C13	−1.5 (2)	C28—C27—Si1—C21	52.66 (17)
C10—C11—C12—C19	177.13 (15)	C28—C27—Si1—C24	−73.87 (17)
C11—C10—C15—C16	90.2 (2)	C29—C27—Si1—C20	−65.74 (16)
C11—C12—C13—C14	1.9 (2)	C29—C27—Si1—C21	−179.69 (15)
C12—C13—C14—C9	−0.2 (2)	C29—C27—Si1—C24	53.77 (17)

### Hydrogen-bond geometry (Å, º)

Cg is the centroid of the C9–C14 ring.

**Table:**

*D*—H···*A*	*D*—H	H···*A*	*D*···*A*	*D*—H···*A*
C25—H25*B*···*Cg*^i^	0.96	2.98	3.699 (3)	132

Symmetry code: (i) −*x*+1, −*y*+1, −*z*+1.
