# Supplementary figures and images for: Crystal structure of ({4-[(4-bromo­phen­yl)ethyn­yl]-3,5-di­ethyl­phen­yl}ethyn­yl)triiso­propyl­silane
Source: Acta Crystallogr E Crystallogr Commun. 2015 Apr 18;71(Pt 5):o321–2. doi: 10.1107/S2056989015007252 (PMC4420139; doi:10.1107/S2056989015007252)

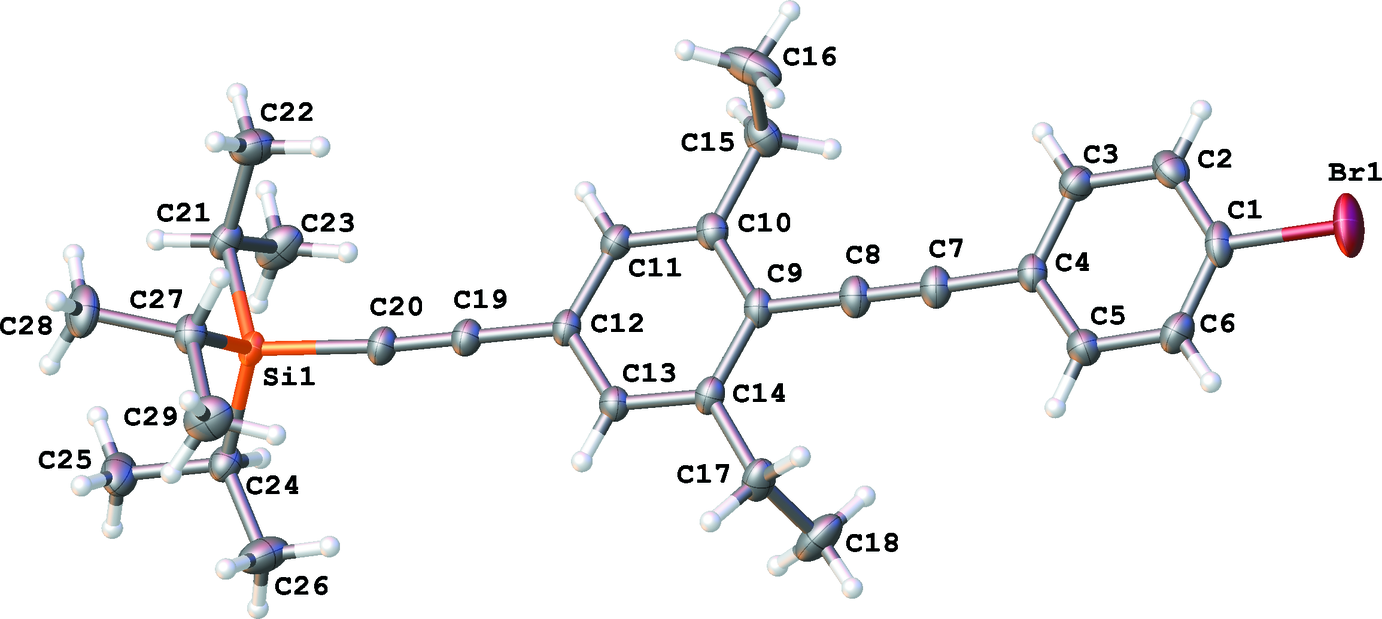

Supplement: Supplementary file 4 [file e-71-0o321-fig1.tif]

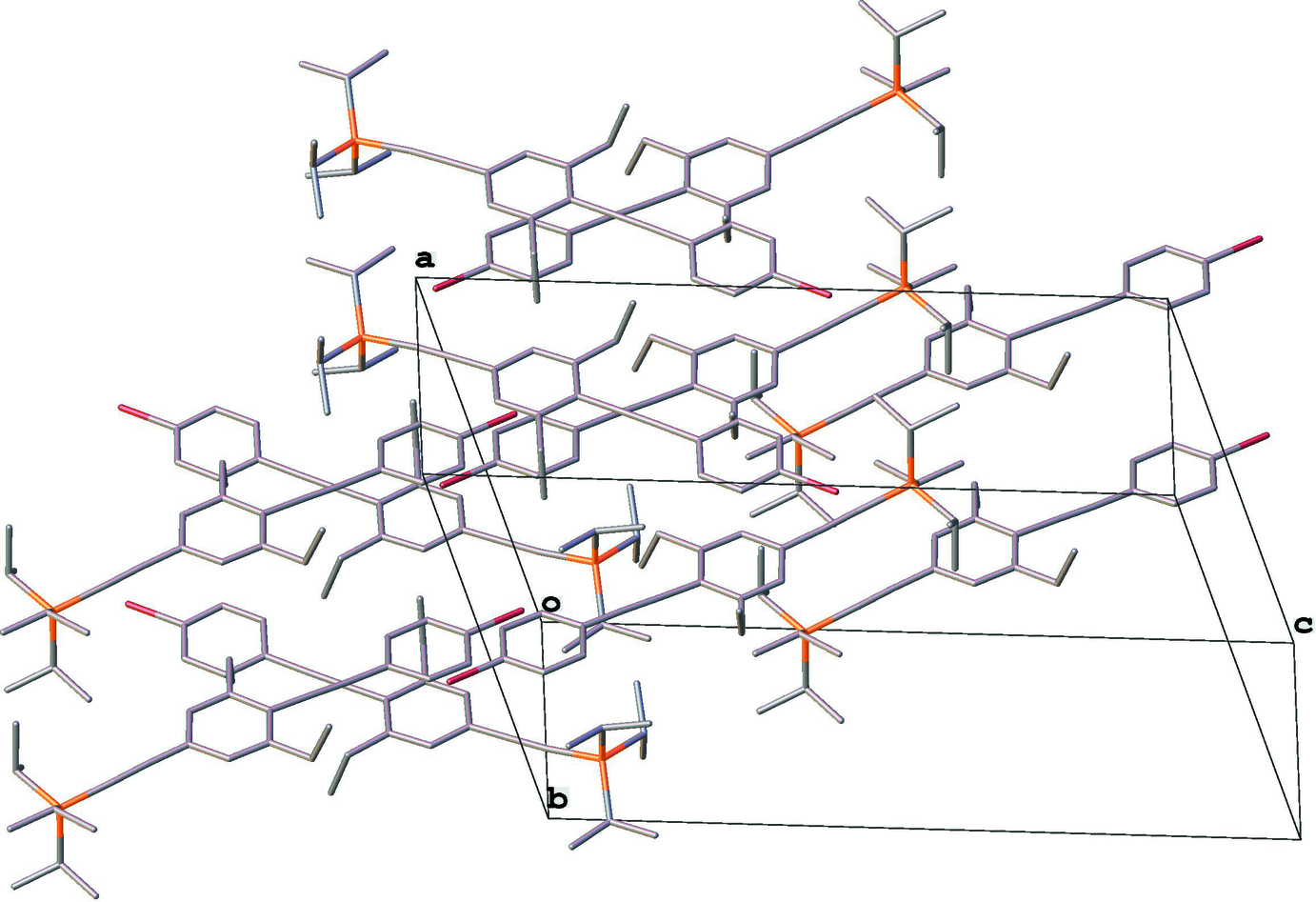

Supplement: Supplementary file 5 [file e-71-0o321-fig2.tif]

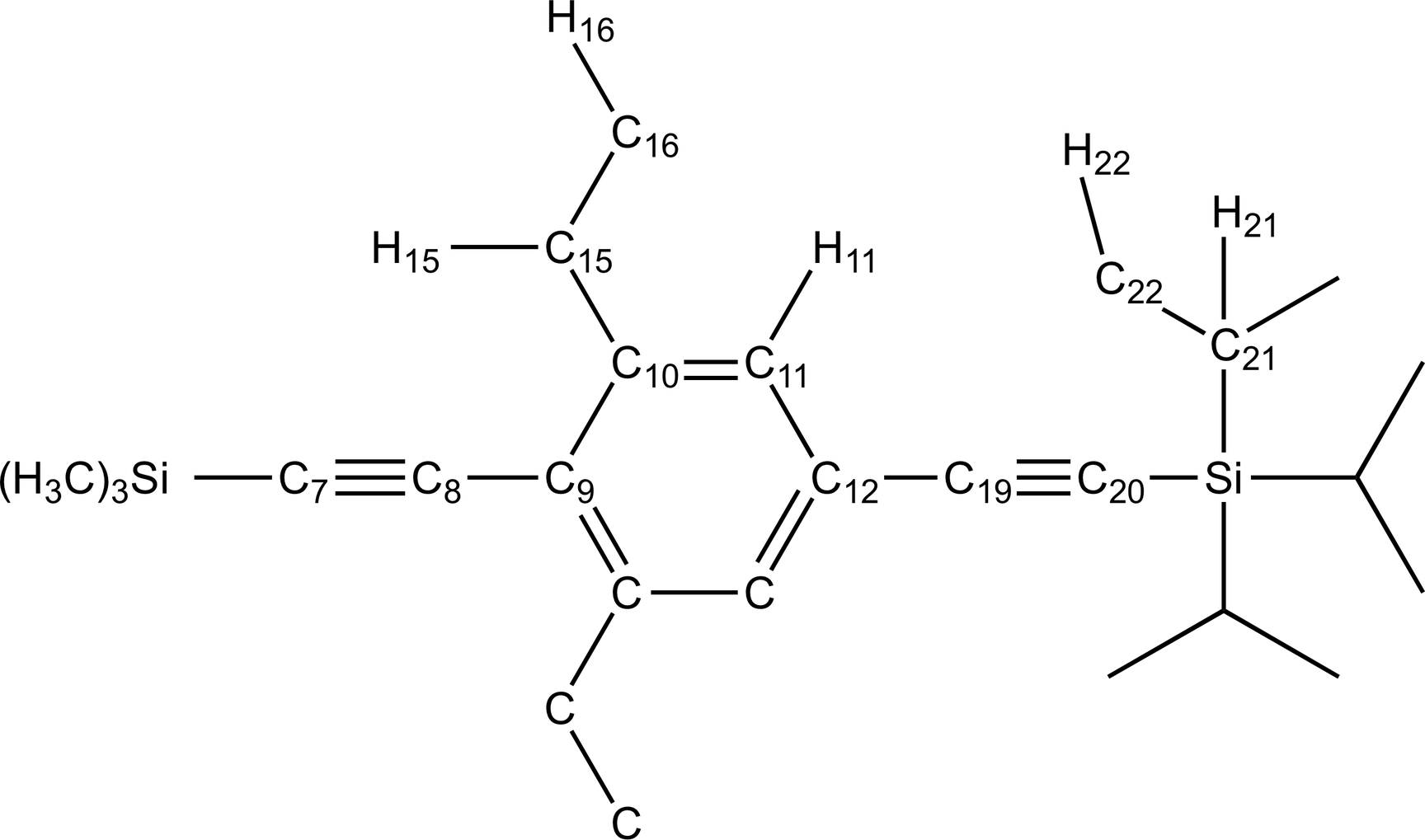

Supplement: Supplementary file 6 [file e-71-0o321-fig3.tif]

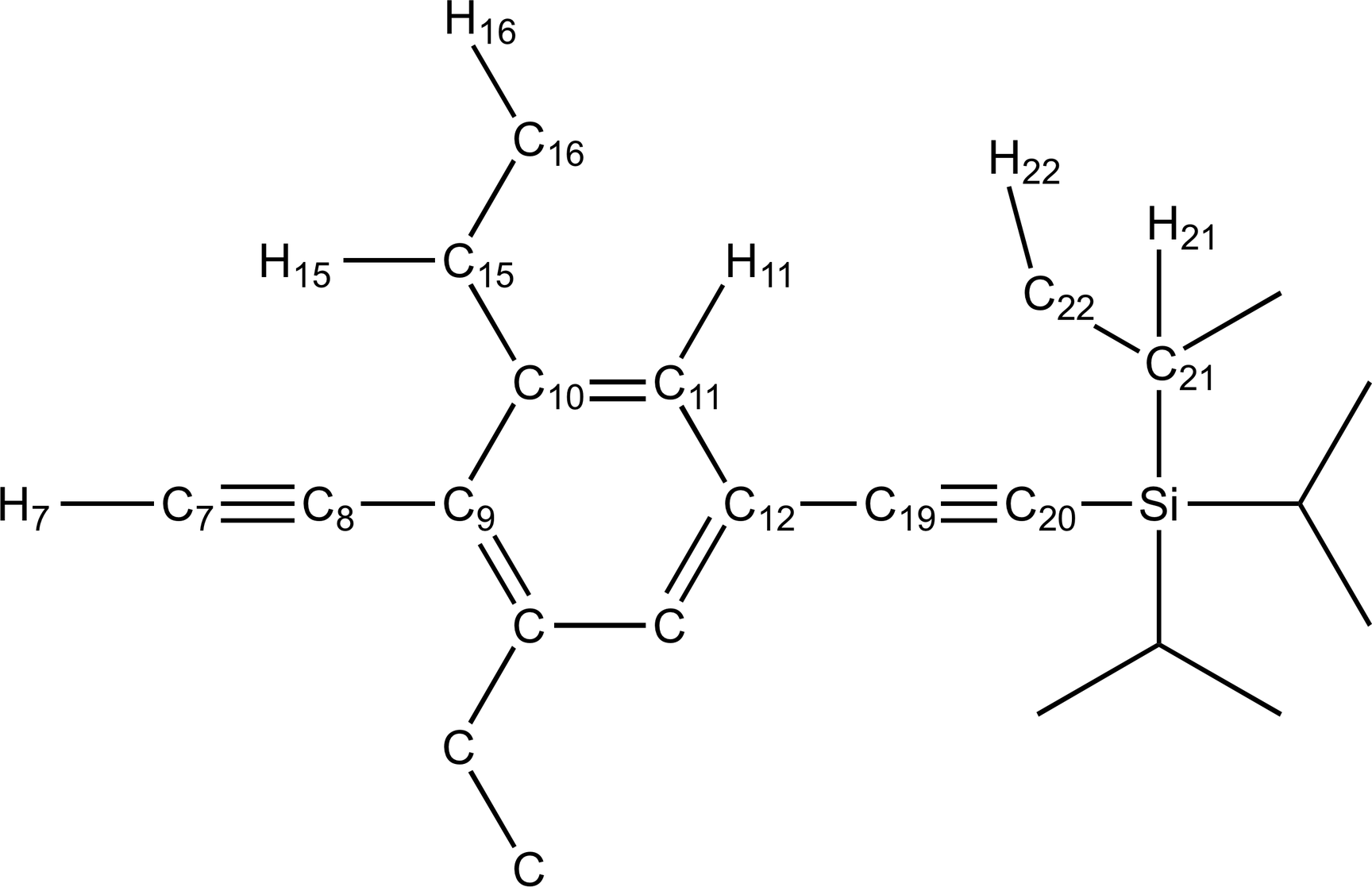

Supplement: Supplementary file 7 [file e-71-0o321-fig4.tif]

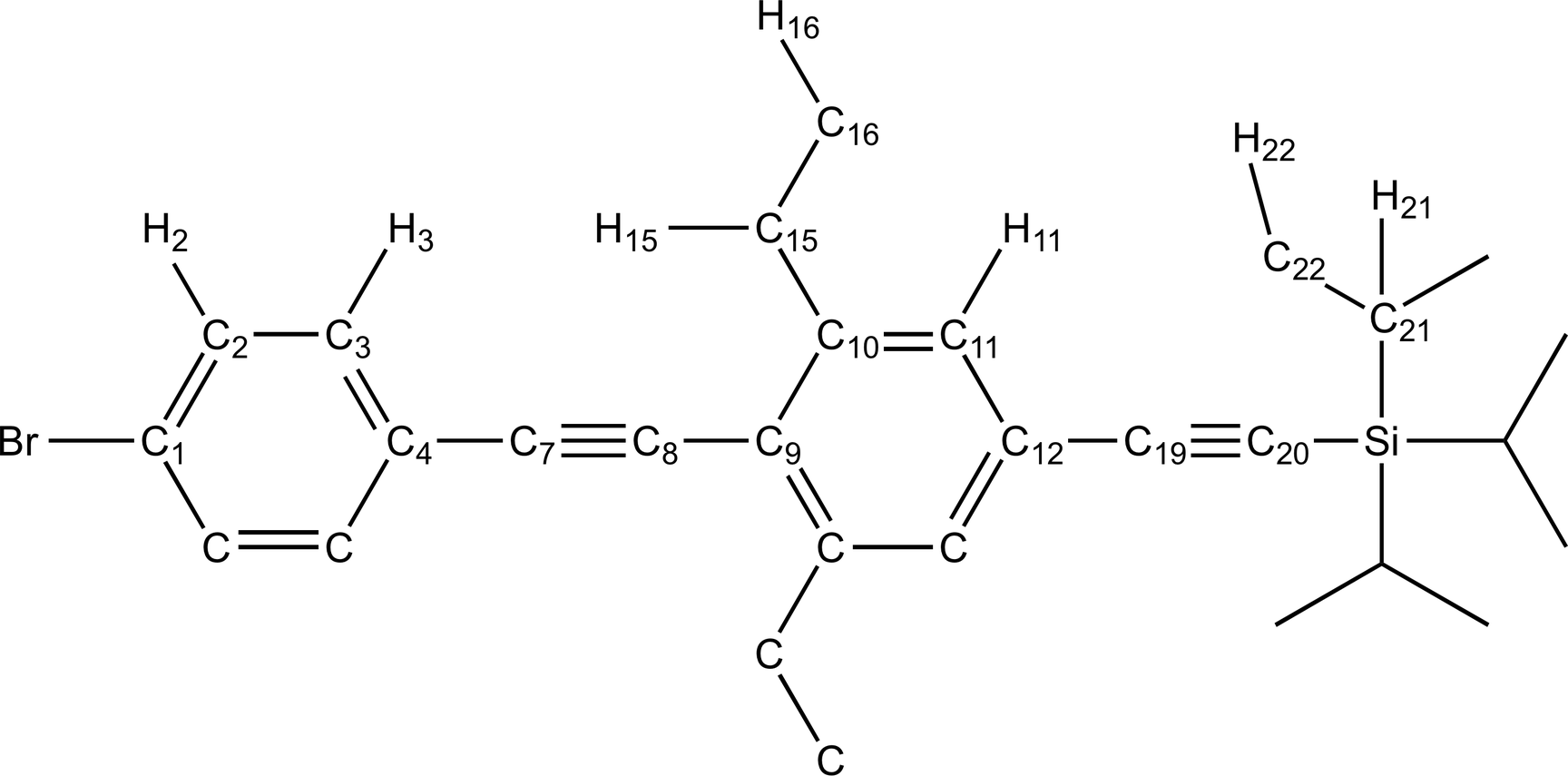

Supplement: Supplementary file 8 [file e-71-0o321-fig5.tif]
